# Experimental and Numerical Study of the Elastic SCF of Tubular Joints

**DOI:** 10.3390/ma14154220

**Published:** 2021-07-28

**Authors:** Mostafa Atteya, Ove Mikkelsen, John Wintle, Gerhard Ersdal

**Affiliations:** 1 Department of Mechanical and Structural Engineering and Materials Science, Faculty of Science and Technology, University of Stavanger, 4021 Stavanger, Norway; ove.mikkelsen@uis.no (O.M.); gerhard.ersdal@uis.no (G.E.); 2 Department of Mechanical Engineering, Faculty of Engineering, University of Strathclyde, Glasgow G1 1XQ, UK; john.b.wintle@gmail.com

**Keywords:** fatigue, offshore structures, experimental testing, tubular joints, hot spot stress (HSS), stress concentration factors (SCF), finite element analysis (FEA)

## Abstract

This paper provides data on stress concentration factors (SCFs) from experimental measurements on cruciform tubular joints of a chord and brace intersection under axial loading. High-fidelity finite element models were generated and validated against these measurements. Further, the statistical variation and the uncertainty in both experiments and finite element analysis (FEA) are studied, including the effect of finite element modelling of the weld profile, mesh size, element type and the method for deriving the SCF. A method is proposed for modelling such uncertainties in order to determine a reasonable SCF. Traditionally, SCF are determined by parametric formulae found in codes and standards and the paper also provides these for comparison. Results from the FEA generally show that the SCF increases with a finer mesh, 2nd order brick elements, linear extrapolation and a larger weld profile. Comparison between experimental SCFs indicates that a very fine mesh and the use of 2nd order elements is required to provide SCF on the safe side. It is further found that the parametric SCF equations in codes are reasonably on the safe side and a detailed finite element analysis could be beneficial if small gains in fatigue life need to be justified.

## 1. Introduction

Fixed steel offshore structures (jackets) are framed structures of tubular members welded together. These are the most common type of offshore substructure for oil and gas exploration and to an increasing degree also being used for offshore wind turbines. The structures are exposed to cyclic wave and wind loads in corrosive environments and fatigue is one of the main design criteria. The fatigue life assessment of welded joints is typically based on S–N curves in combination with a damage rule. The assumption of linear cumulative damage using the Palmgren–Miner rule is widely applied [1].

Design S–N curves for various classes of welded joints have been established primarily based on test specimens in the laboratory [2]. Due to the geometry of welded tubular joints, high-stress gradients exist in the transition zone between the weld line and the base material, and in linear stress analysis, the geometric discontinuity of the weld toe defines a stress singularity.

**Figure 1:**
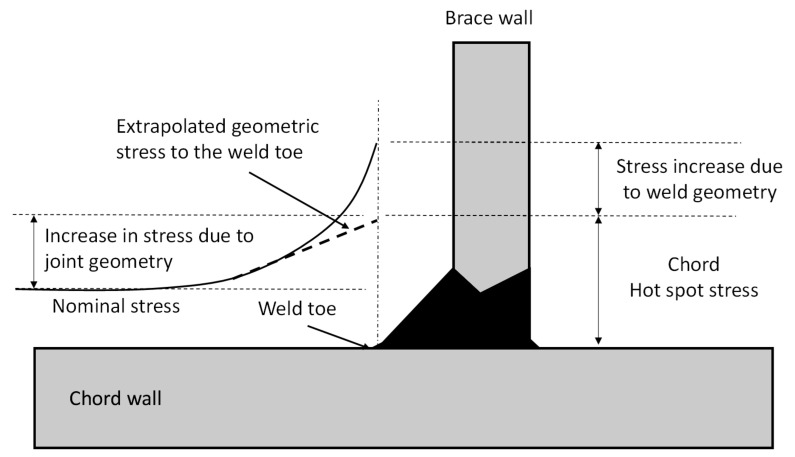
Definition of tubular joint stresses.

In general, stresses in tubular joints arises from three main causes, excluding the residual or misfit stresses as shown in Figure 1; these are the nominal stresses: stresses in the members under applied external loads without considering the detail of the joint intersection, i.e., the framing action of the jacket structure under applied external loads. Hot spot stress (deformation stresses): stresses close to the weld toe arising from the deformation of the tubular wall to maintain continuity at the intersection with the weld profile under the applied external loads. Finally, notch stress: stresses introduced due to the geometrical discontinuity at the weld toe/root.

In stress analysis, hot-spot stress is the highest value of stress that can be inferred as near the weld as possible, generally at the weld toe. It can be estimated by modifying the nominal cyclic stress arising from the loads in the joint by a stress concentration factor (SCF). The SCF is intended to incorporate the stress of the local joint geometry and the discontinuity but omits the influence of the weld toe itself.

In practice, the tubular joint SCF is usually calculated from parametric equations given in standards such as ISO 19902 [1], API RP2A [3] and DNVGL-RP-C203 [4] or from case-by-case finite element analysis. The standard and code-based SCFs are expected to provide upper-bound values for use in design and life extension assessments.

Various parametric formulas, expressed with non-dimensional joint parameters for different types of loading and boundary conditions, have been developed both experimentally and numerically. Beale and Toprac [5], used small scale T and KT joints under tensile loading. Reber [6] and Visser [7] attempted to estimate the hot-spot stress of T, Y and K joints under compression loading using finite-element programs. Marshall [8] adopted the Kellogg [9] formula to express SCF for brace and chord of K joints using classical solution methods. Kuang et al. [10] used finite element analysis to develop semi-empirical formulae to cover the SCF on brace and chord side for 138 T, Y, K and KT joints. Gibstein [11] carried out parametric analysis for seventeen T joints using FE analysis to investigate rigidly fixed chord ends. Wordsworth and Smedley [12] presented one of the first comprehensive parametric formulae for SCFs of T,Y, KT and DT joints. The parametric study was obtained from acrylic model testing. Later Efthymiou [13] provided generalised influence functions developed for use in fatigue analysis. The SCFs are derived by establishing influence functions describing the ‘hot spot’ stress at a particular location of a specific member. It has been developed by performing finite element analyses using the in-house PMBSHELL program. The program uses thick shell elements for modelling the members and 3-D brick elements for the weld. The influence functions by Efthymiou are implemented in codes and standards and are the most widely used currently.

Smedley and Fisher [14] concluded that since Efthymiou SCFs are design formulae giving a mean fit to his FE database, it tends to underpredict in 20–40% of the cases compared to Lloyd’s Register experimental database consisting of steel and acrylic models. Hence, the first impression of this indication could be that Efthymiou formulae provide SCF values on the unsafe side of the database. However, ISO 19902 [1] indicates that Efthymiou’ parametric formulae have a bias of 19% to the safe side compared to the experimental values, with a coefficient of variation (CoV) of 19% (20% according to DNVGL-RP-C210 [15]). The formulae have been accepted as providing a reliable design basis for structures with tubular joints.

The parametric equations have been typically developed from extrapolated experimental strain gauge measurements or generic finite element analysis of representative test joints. Although case-by-case finite element analysis methods use linear extrapolation from the same points typically used to place strain gauges in experiments, this approach overlooks the weld profile tolerances captured in tests. As a result, the analyst cannot normally incorporate the as-built weld profile into a model accurately. Thus, in some cases, the numerical finite element values could underestimate the SCF compared with those derived from parametric equations and experiments.

This paper presents a review and comparison between case-by-case finite element analysis and experimental and parametric estimates of SCF for cruciform tubular joints (also called double T or X joints based on the specific structural behaviour) with geometric parameters of *β* = 0.5, *τ* = 1 and *θ* = 90° under axial loading.

Experimentally determined SCFs were derived from multiple strain gauge measurements made on four test specimens loaded in a fatigue rig. Stress concentration factors are obtained at four points along the joint periphery from the chord saddle to the chord crown. These were compared with results from finite element models with idealised weld profiles and models with weld profiles matched to the test specimens and with the values calculated from the standard parametric equations.

## 2. Design Stress Concentration Factor (SCF)

This section presents a comparison between the available formulae to estimate the SCFs of a DT joint then compare it to the existing database. Finally, it provides an estimate for the SCFs distribution along the brace-to-chord intersection from design SCFs and previous experimental work.

### 2.1. Existing Parametric Formulae

Two parametric equations do exist to estimate the chord saddle SCFs of DT joints, namely, Wordsworth/Smedley (1) and Efthymiou (2).

(1) $${\mathrm{SCF}}_{\mathrm{chord}\;\left(\mathrm{saddle}\right)}=1.7\beta \gamma \tau \left(2.42-2.28\beta^{2.2}\right)\sin^{\beta^{2}\left(15-14.4\beta \right)}\theta$$

(2) $${\mathrm{SCF}}_{\mathrm{chord}\;\left(\mathrm{saddle}\right)}=3.87\gamma \tau \beta \;\left(1.1-\beta^{1.8}\right)\;{\left(\sin\theta \right)}^{1.7}$$

where β=d/D, γ=D/2T and τ=t/T;

*t* brace wall thickness;
*T* chord wall thickness;
*d* brace outside diameter;
*D* chord outside diameter;
θ is the angle between the brace and the chord.

**Figure 2:**
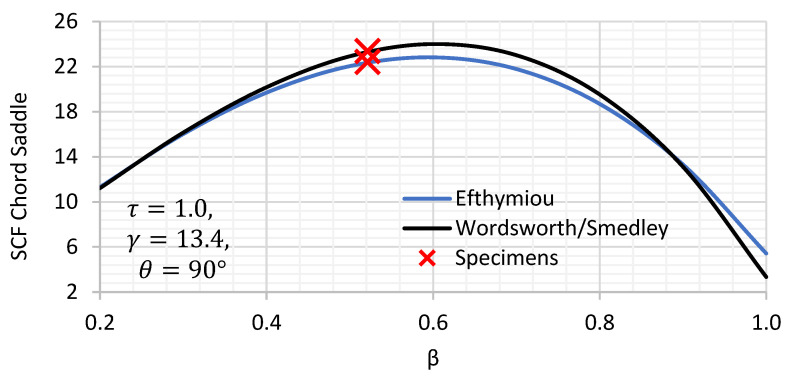
Axially loaded DT joints: chord SCF variation with β for a set of joint parameters (θ, γ and τ ).

**Figure 3:**
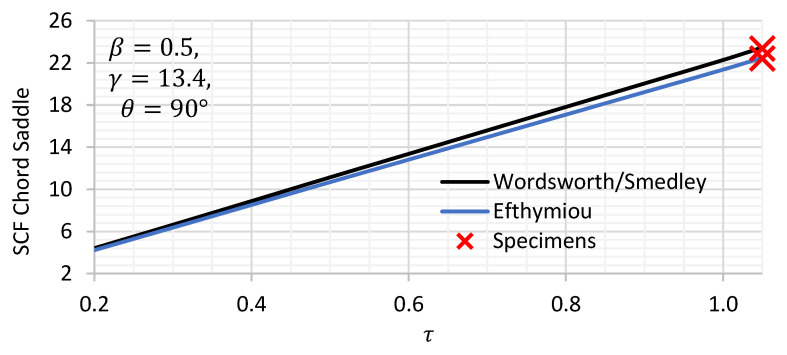
Axially loaded DT joints: chord SCF variation with τ for a set of joint parameters (θ, γ and β ).

A comparison of the variation in the chord saddle SCF for β and τ between the two sets of formulae are given in Figure 2 and Figure 3. The expression of chord saddle from Efthymiou and Wordsworth/Smedley showed that both expressions have slight to no difference. Under constant τ, Wordsworth and Smedley’s expression show higher SCF by some 2% than Efthymiou for midrange β values while Efthymiou shows higher values than Wordsworth and Smedley at β higher than 0.9. Under constant β, Wordsworth shows higher values than Efthymiou by 3% for any τ value.

### 2.2. Comparison with Previous Experimental Results

There is a limited amount of published data on the SCFs of DT tubular joints, which can be compared to Efthymiou and Wordsworth and Smedley parametric equations. However, some published data collected from the literature [16,17,18,19] are included in this comparison.

**Figure 4:**
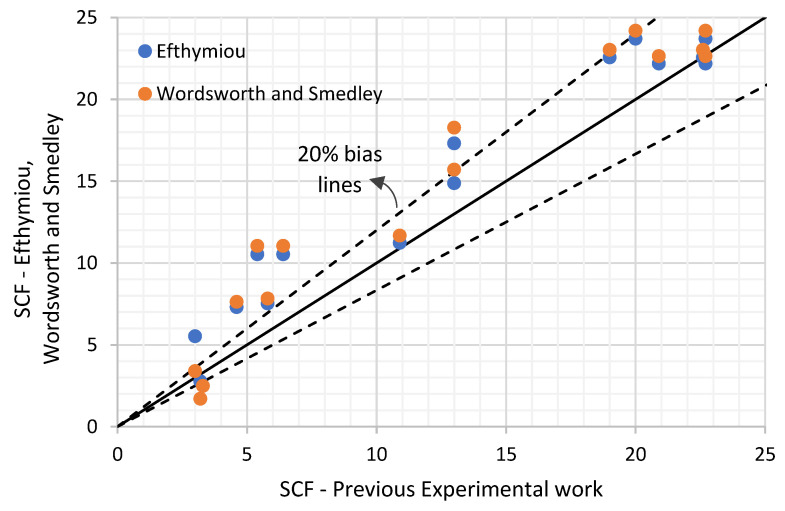
Existing database for DT joints under axial loading.

A comparison of the published data is shown in Figure 4 (within the validity range as per the codes and standards). In this comparison it was found that Efthymiou data shows a bias of 20% on the safe side while the Wordsworth and Smedley SCF estimate shows a bias of 22% on the safe side, reasonably in line with the findings of ISO 19902 [1] mentioned earlier. Hence it can be concluded that Efthymiou provides a closer estimate to the real SCFs than Wordsworth and Smedley except for cases with β higher than 0.9.

### 2.3. SCFs Distribution at the Brace-to-Chord Intersection

Efthymiou provides SCFs for the saddle and crown point of a tubular joint. For joints under pure axial loading, linear interpolation is then recommended to estimate stresses at points between the saddle and crown [20]. Table 1 shows the estimated SCFs from the chord saddle to crown based on design Efthymiou SCFs and data from previous experimental work.

## 3. Experimental Stress Concentration Factor (SCF)

This section presents the stress distribution at the brace/chord intersection and SCFs from present experimental work. It is a part of experimental testing to study crack arresting techniques in tubular joints. The testing program for the specimens is to undergo cyclic loading until precracking (developing a through-thickness crack), then crack arresting by means of hole drilling followed by cyclic reloading until failure. Strain gauges are mounted on the specimen to map the stresses at the joint intersection and estimate the SCF during all the fatigue loading stages. The SCFs of interest in this paper are estimated from the initial load before commencing the precracking stage.

### 3.1. Specimen Design

**Figure 5:**
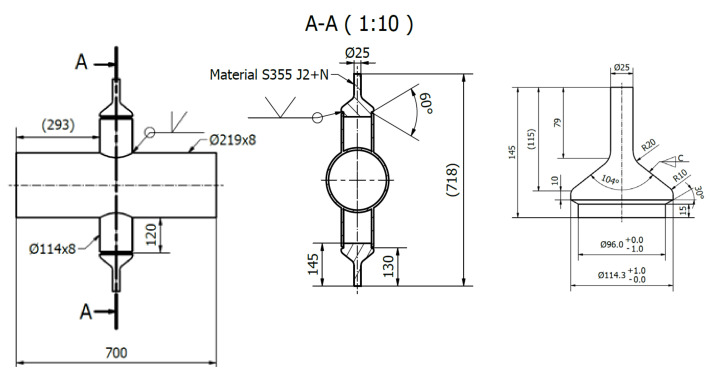
DT Joint geometry (all units are in mm).

Four DT joints fabricated with dimensions of 219.1 mm outer chord diameter (D) with 8.2 mm chord wall thickness (T) and 114.3 mm brace outer diameter (d) with 8.5 mm brace wall thickness (t) with geometric factors of β=d/D=0.5, τ=t/T=1 and chord to brace angle θ of 90° as shown in Figure 5.

All the steelwork fabrication, inspection and testing were done in accordance with DNV-OS-C401 [21]. The welded specimen underwent a detailed dimensional and visual inspection to ensure that all the dimensions and welded details are within specifications.

### 3.2. Methodology for Determining Stress Concentration Factors

**Figure 6:**
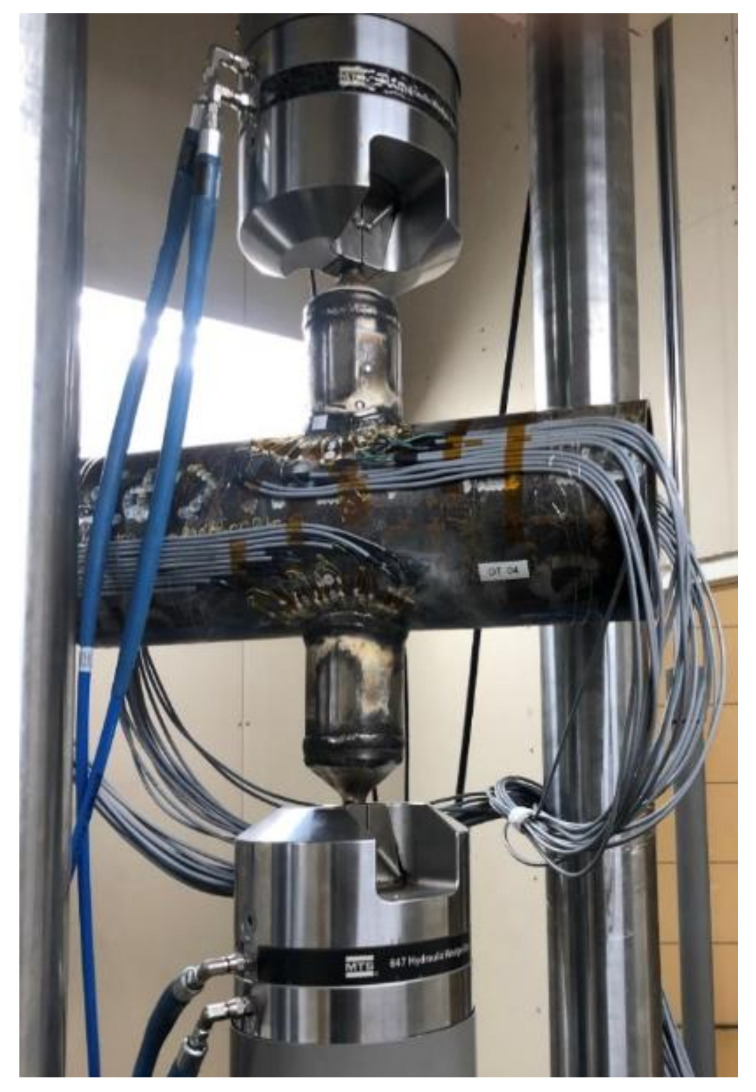
Test specimens setup.

The distribution of stresses at the brace-to-chord intersection is measured by strain gauges perpendicular (HBM, Darmstadt, Germany) to the weld toe for all the specimens under tensile axial loading. Test Specimens setup are presented in Figure 6.

The stress concentration factor (SCF) or strain concentration factor (SNCF) are defined as the ratio of the hot spot stress or strain to the calculated nominal brace stress or strain. Hot spot stresses can be determined by extrapolating stresses from the given points to estimate the stress at the weld toe. This approach distinguishes the hot-spot (deformation) stress from the notch stress and uses the given points for measurements outside the notch region. Three methods of extrapolation were adopted in a study by the department of energy [22] to determine the hot spot stress to calculate the SCF subsequently:

(1) Method A: Linear extrapolation of maximum principal stresses.
(2) Method B: Curvilinear extrapolation of maximum principal stresses.
(3) Method C: Curvilinear extrapolation of strains perpendicular to the weld toe.

The study concluded that the curvilinear extrapolation of maximum principal stresses was 10% higher than the linear method. In later studies [22,23,24], it was determined to use method A, the linear extrapolation, as the method of ‘hot spot’ SCF determination to be used with the S–N curves for tubular joints. The method was adopted in the current codes and standards.

### 3.3. Loading Procedure

Nominal stresses were estimated by measuring the load applied on the brace from the rig built-in load cell and the brace’s cross-sectional dimensions, which were measured to an accuracy of 10^−2^ mm.

Fifty percent (50%) of the yielding strain was applied for 100 sinusoidal load cycles to a strain of approximately 700 microstrains at the chord saddle to ‘shake down’ the strain gauges. After ‘shake down’, the specimens were loaded for 200 cycles for a hot spot stress range of approximately 280 MPa with a load frequency of 3 Hz. Based on 200 Hz readings the data was interpolated to determine average response range for the estimation of SNCFs. This technique was applied to stabilise the response in the joint and to minimize eventual data acquisition errors.

### 3.4. Strain Gauge Locations, Data Logging and Processing

**Figure 7:**
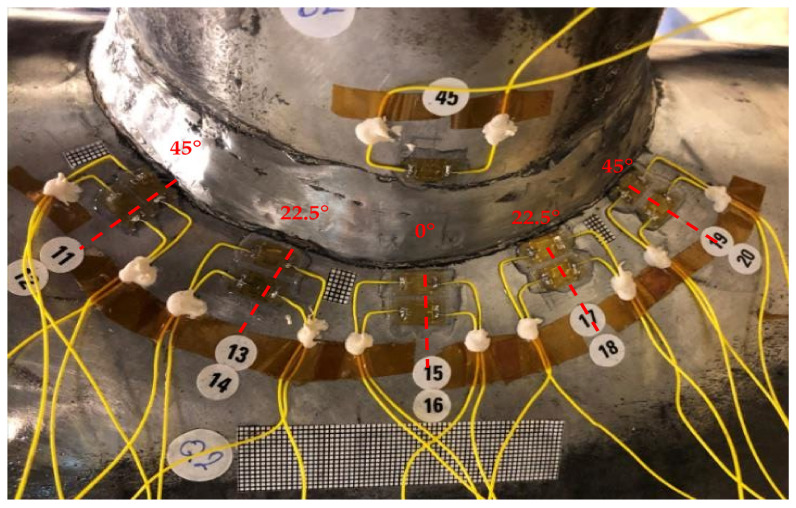
Chord saddle strain gauge layout.

The strain gauges were glued to the chord and brace members to determine the stress distribution on the chord side from the saddle to the crown and nominal stresses in brace members. The chord saddle strain gauges layout is shown in Figure 7. The method of extrapolation used for each specimen tested was the linear extrapolation method. Strain gauge locations were placed based on the recommendation from the UKOSRP [25,26,27] project. The strain gauge locations to be located outside the notch region, with the first point defined as the greatest of 0.2$\sqrt{rt}$ and 4 mm. The second point was located at the brace-to-chord intersection according to the location:

(1) Chord saddle = πR/36;
(2) Chord crown = $0.4\sqrt[{4}]{rtRT}$;
(3) Brace side = $0.65\sqrt{rt}$.

where *r* is the brace outer radius and *R* is the chord outer radius.

Based on the recommendations above, the first row of strain gauges on the chord side was intended to be glued with its centre located at 4.42 mm from the weld toe, while the second row of gauges was intended to be glued with a varying distance of 9.56 mm from the weld toe at the chord saddle and 10.29 mm from the weld toe and chord crown.

Due to the space limitation on the chord saddle, only linear strain gauges perpendicular to the weld toe were glued and lateral strain gauges were omitted. The strains measured as-is from this layout was not sufficient to calculate the maximum principal stresses. A study by Lloyd’s Register tested three ways of calculating the SCF from the strain gauge results [28]:

(a) Extrapolation of maximum principal stresses.
(b) Extrapolation of the strains perpendicular to the weld toe.
(c) Conversion of the SNCF in method (b) to biaxial stress.

The study [28] found that for 90° joints, the maximum principal stress estimated with method (a) was approximately 15% larger than the directional stresses based on method (b).

This study applies experimental strain measurements combined with FE analysis to estimate the principal stresses. The measured strains perpendicular to the weld toe are presented in Table 2 and used to validate the FE analysis. The analysis was performed using linear and quadratic elements with the linear extrapolation method to estimate the SCFs based on directional and maximum principal stresses. The range of results from FE analysis are shown in Table 2.

The FE analysis showed that the principal stresses at the saddle were 14.7% higher than the perpendicular stresses and increased slightly towards the crown, reaching 17.6%. The results are in line with the study by Lloyd’s Register [28]. In the present work, the correction factors as presented in Table 2 were used to estimate the principal stresses at different locations from the experimentally measured strains perpendicular to the weld toe. Details of the FE analysis are presented in Section 4.

### 3.5. Experimental Work Results

The experimental work presented herein followed method b, as described in Section 3.4, for the SCF extrapolation from strain gauges. Hence, the SCFs from the experimental work were updated by correction factors as per Table 2 to be compatible with the parametric code equations (Efthymiou formulae [13]) and the SCFs extracted from FE models.

**Figure 8:**
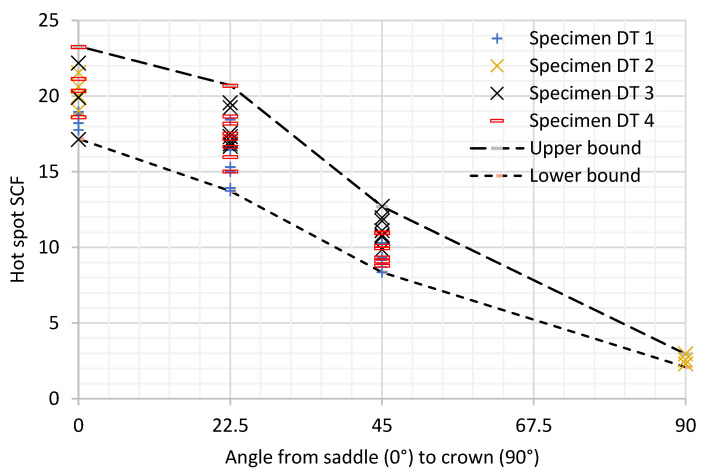
Experimental hot spot SCF along the weld toe from the saddle (0°) to crown (90°).

The distribution of stresses along the circumference of the brace to chord intersection as measured by linear extrapolation of principal stresses for all the specimen tested are presented in Figure 8 and Table 3. The distribution was plotted along the intersection with reference to an angular position from the saddle at 0° to the crown at 90°.

**Figure 9:**
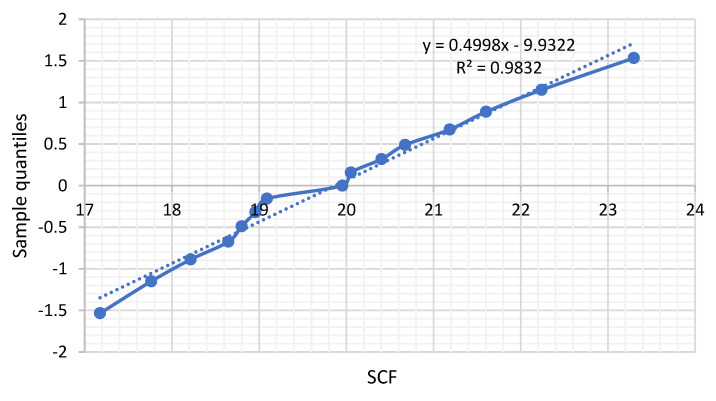
SCF values from experimental work plotted in a normal distribution paper, indicating a mean value of the SCF of 19.87 and a standard deviation of 2.

The distribution of the SCF’s at the saddle point from the experimental work fit reasonably well the normal distribution, as shown in Figure 9. By bootstrapping the data, it is indicated that the 90% confidence interval for the mean value of the SCF was [19.3, 20.6] and for the standard deviation [1.2, 2.1], providing a CoV in the range of 5–10%.

### 3.6. Review of the Accuracy of Experimental SCFs

**Figure 10:**
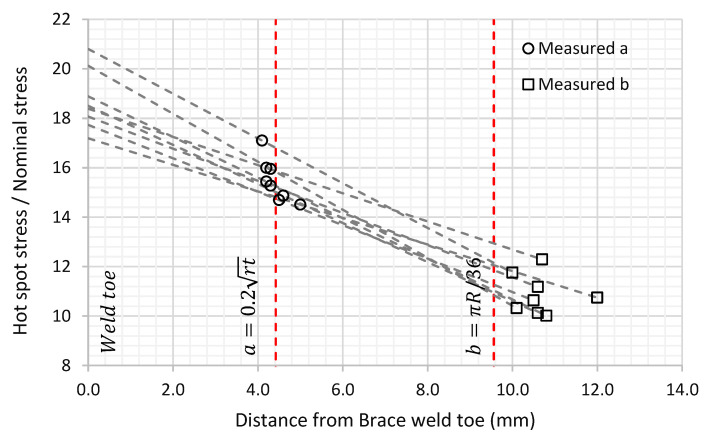
Experimentally measured SCFs sensitivity.

The precision in the strain gauge’s locations was studied at eight locations of the chord side saddle from two randomly selected specimens of the total of four. The variation between actual and average strain gauge location is a useful indicator of the overall accuracy of the SCF for the specimen. The actual strain gauge’s locations to the weld toe were measured to an accuracy of ±0.1 mm, then the measured points and their strain readings were used to extrapolate for the SCFs linearly. The results show that the location of the strain gauges at point “a” were scattered without evidence of any systematic error. However, for the strain gauges at point “b” there is a scatter around a mean shift of 1.1 mm from the intended “b” location. The shift in mean value can be explained by the length of the strain gauge carriers. The carrier’s length was 5 mm while the difference between point “a” and “b” was only 5.1 mm, which made it challenging to manually position the strain gauges spot on. Figure 10 shows the distribution of strain gauges along the weld toe’s intended “a” and “b” locations and the extrapolation lines to the SCF value for each pair of points. After strain gauges installation, the average distance between “a” and “b” was approximately 6.3 mm.

The average SCF measured at the chord saddle from these eight points was 18.71 with a standard deviation of 1.21, providing a CoV of 6.5%, while the uncorrected average for the same two specimens provides a mean SCF of 19.4 with a CoV of 8.4%. The difference between the mean value between these two samples can be assumed to be due to inaccuracy in the strain gauge locations, while the variation between the results from the eight points was a combination of other inherent uncertainty (geometry, material, measurement, etc.).

This implies that the shift in strain gauge location introduces a bias of 3.7% to the safe side, which can also easily be seen to be reasonable by a geometric evaluation. For the entire sample, the average SCF was found to be 19.87 with a standard deviation of 1.72 and CoV of 8.7%. To adjust for the strain gauge location, assuming the same trend for the two remaining specimens as for the two tested, the mean value of the SCF should be set to 19.87/1.037 = 19.16 and a CoV of 8.4%.

In summary, it can be concluded from the obtained statistical coefficients that the average test values were in good agreement with the actual values and the data can be treated as valid. The test data was further verified by correlation with the finite elements and available parametric formulae.

## 4. Finite Element Analysis Based SCFs

This section presents finite element analysis to estimate SCFs of DT joints considering the effect of the weld profile variation and the methodology of modelling on the SCFs results.

### 4.1. Modelling and Analysis Approach

The DT joint consists of a 219.1 mm × 8.2 mm member with an adjoining 114.3 mm × 8.5 mm member. The geometry is considered to reflect a single-sided weld made from the outside. During the fabrication, the joint braces were cut so that the brace inner circumference matched the chord profile. The braces were then joined to the chord with single-sided welds.

### 4.2. Weld Profile

**Figure 11:**
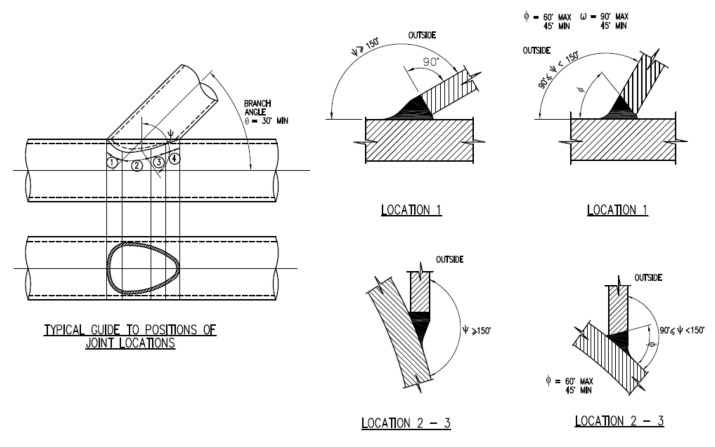
Weld profile specifications as issued to the fabricator for these tests in line with appropriate standards, see Section 4.4.

The weld details were as per the weld preparation instructions shown in Figure 11. The weld at the saddle was assumed to be of weld location 2, as shown in the weld details drawing and the weld at the crown was assumed weld location 1.

**Figure 12:**
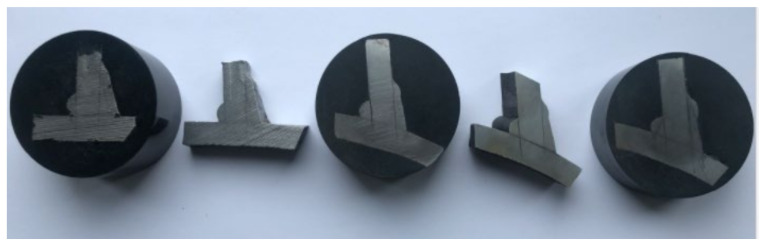
Actual weld profile cut from one of the specimens.

Specimen DT1 was cut into segments post-testing each 20° from the saddle to the crown to measure the weld profile’s cross-section at the brace-to-chord intersection accurately. The shape of the weld profile modelled was based on the measured cut segments shown in Figure 12. At the saddle, the brace was cut back to normal to the axis of the brace cylinder. At the crown, the parent material was assumed to be cut back to approximately 45° and then filled so that the intersection between the weld and the brace was at a right angle. The weld profile was a smooth transition based on an elliptical profile between the cut segments.

The weld toe and root radii were omitted for simplicity. They were unlikely to affect the resulting hot spot stress as this was extrapolated from points sufficiently away from the sharp corners.

### 4.3. Model Description

**Figure 13:**
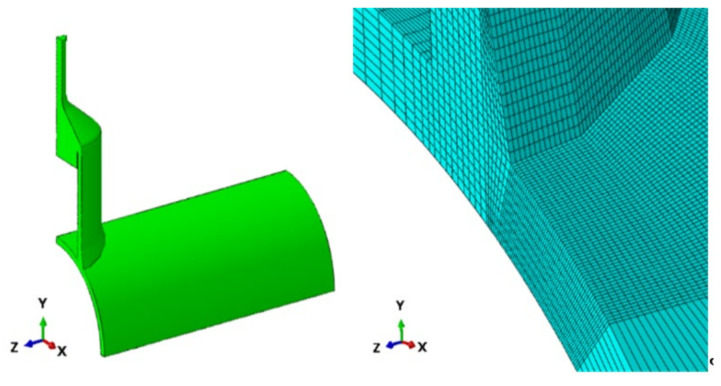
Model and mesh idealisation.

The 3D model was developed using Dassault Systems Abaqus [29]. The DT joint was modelled using first order C3D8R and second order C3D20R hexahedron elements for the joint stubs with a characteristic element size of 1 mm at the chord to brace intersection. This mesh size provided nine elements through-thickness. A coarser representation of the outer extents of the tubular members was created using the same elements to a distance of approximately 40 mm from the weld (see Figure 13). The end cone was modelled at the brace ends to capture as accurately as possible to the actual joint behaviour. The joint was symmetric about the three principal planes. Only one-eighth of the joint was modelled. The saddles occurred at 0° and the crowns at 90°. All material (parent and weld filler) was linear elastic with a Young’s modulus of 207 GPa and Poisson’s ratio of 0.3. In order to generate the required SCFs, the joint was loaded in the axial directions. A symmetric (balanced) response was achieved by loading the quarter rod at the end cone.

### 4.4. SCF Calculation Methodology

Standards and recommended practices provide two methodologies for SCF calculation. The first is the direct extraction of stresses for use in fatigue calculations, where stresses are measured from a strain gauge (grid length of 3 mm) placed perpendicular to the weld toe at a distance within 6 mm to $0.1\sqrt{rt}$. This method is adopted in API RP 2A [3] and AWS D1.1 [30]. The second method is the linear extrapolation of principal stresses. This method is recommended by the UKOSRP [25,26,27] joint industry project.

In the finite element analysis (FEA) of this joint, the first method would imply reading the stress at the Gaussian integration points within a distance between 6 and 2.2 mm. The second method would imply reading the unaveraged principal stresses at both 4.42 mm and 9.56 mm from the weld toe and using these to extrapolate to the weld toe.

The purpose of using the FEA was to accurately compute the stresses at the points of interest. These points of interest need to be aligned with the elements’ integration points, whether the direct extraction or the linear extrapolation method is selected. This can be done by using shell elements, but shell elements are not recommended for complex details and high local bending [31]. The alternative to shell elements is to use solid elements. However, these do not have integration points on the element surface. A possible mitigation for solid elements is the use of dummy membrane elements on the surface that share the same surface nodes. This allows for extraction of the stresses at the integration points in the aligned membrane elements, rather than extrapolating the stresses to the surface of the solid elements. However, it is rather impractical and cumbersome to use such dummy membrane elements and align the integration points’ locations with the points of stress extraction, and a finer mesh without such membrane elements are often a better solution. This will also result in nodal stresses closer to the stresses at the integration points.

In this work, the linear extrapolation method was primarily used to determine stress at weld toe, as this would be equivalent to the experimental work, although results using the direct extraction method is also presented.

**Figure 14:**
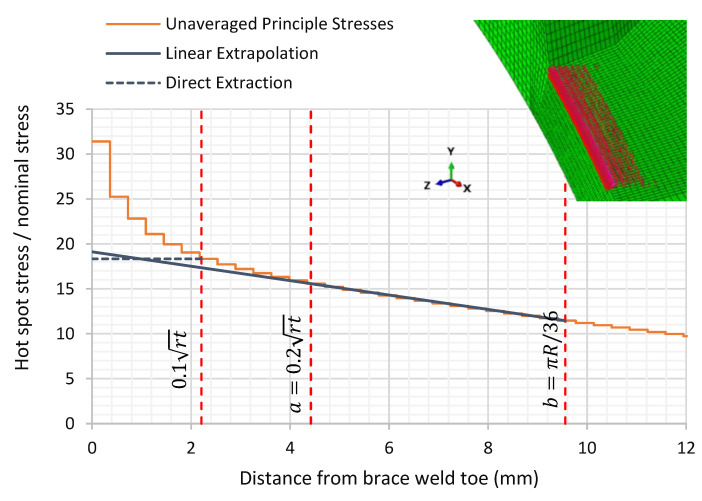
Direct and linear extrapolation of SCF from first-order 8-node brick elements.

In the linear extrapolation method, the principal stresses were extracted at each node on the path perpendicular to the weld toe and these were used to interpolate the stresses at point “a” and “b” (the points of interest). The extrapolation of the SCF at the saddle, from the stresses at these two points, is shown in Figure 14. The unaveraged stresses were used in this study. These were extrapolated from the integration points in the elements.

The SCF for the weld toe was taken as the maximum absolute value of the principal stress divided by the nominal stress. SCFs were calculated along the circumference of the chord side toe at the saddle, 22.5° from the saddle, 45° and at the crown. The plots were taken for both the direct extraction and linear extrapolation methods.

### 4.5. FE Verification and Validation

#### 4.5.1. Mesh Convergence

The weld toe area is a mesh sensitive area as meshing with few elements will result in rapidly varying stresses. Hence, a relatively fine mesh is required. A mesh convergence study was performed first to find the optimum number of elements required to provide reasonable numerical accuracy. The relative convergence method is the only way to confirm that the proper mesh is achieved. The method compares the results from subsequent models where the mesh is systematically refined. Two parameters of mesh refinement were considered in this analysis. The first is the characteristic element length and the second is the number of elements through-thickness. The study was performed for both first-order elements and second-order elements Table 4 and Table 5.

For the first-order elements using linear extrapolation, 3% relative convergence was achieved in the SCF estimation from the averaged principal stresses at an element length of 0.36 mm with nine elements through-thickness. While for the second-order elements, 1.5 mm element length and four elements through-thickness were enough to achieve less than 1% relative convergence.

Reference points for extrapolation can be used as the averaged or unaveraged stress between element nodes. The finer the mesh, the closer the results from both averaged and unaveraged stresses. First-order elements with reduced integration (1 point) at the most refined mesh showed a difference in the linearly extrapolated SCFs of 1.0% between the averaged and unaveraged principal stresses and 1.6% for the direct extraction method. While for second-order elements with reduced integration (8 points), the average and unaveraged stresses for both direct extraction and linear extrapolation showed less than 1% variation.

#### 4.5.2. Results Verification and Validation

**Figure 15:**
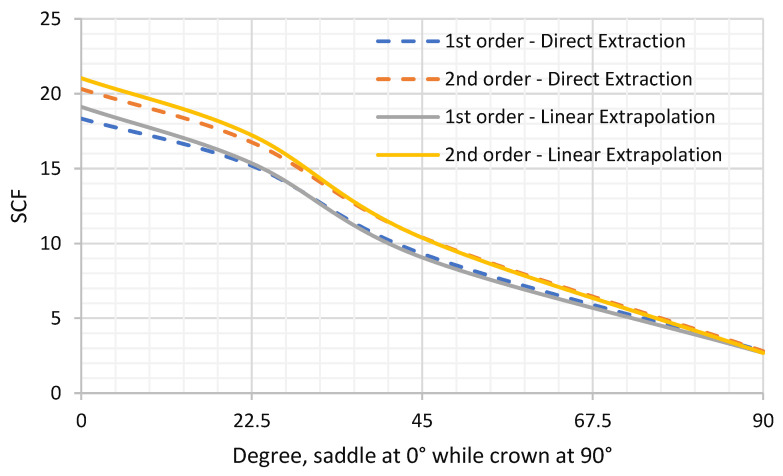
Direct and linear extrapolation of SCFs for first and second-order elements.

**Figure 16:**
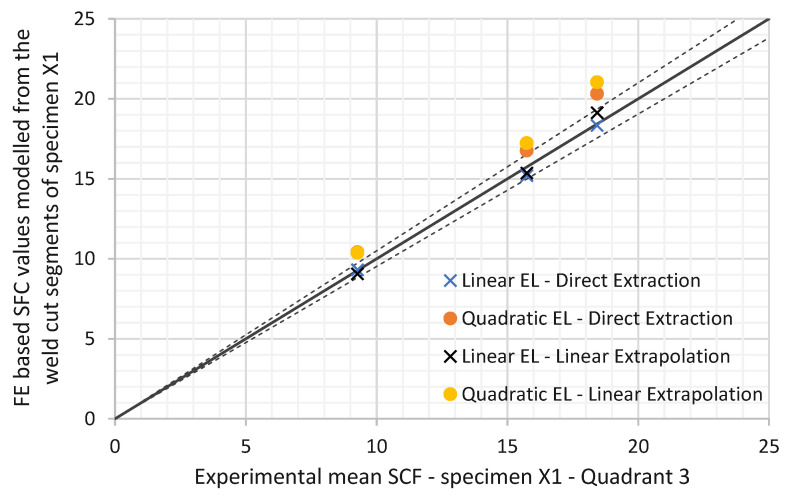
Experimental mean SCF values against FE SCF values.

Two types of elements were tested, first-order 8-node brick and second-order 20-node hexahedron elements. For the same mesh density of 0.36 mm element length, nine elements through-thickness in the vicinity of the weld toe, the circumferential variation of the calculated SCFs along the chord-side and the two SCF calculation methods are provided below in Figure 15 and Figure 16 and Table 6. It can be shown that second-order elements consistently provided higher SCF than linear elements by 10% at the saddle location, then decreased circumferentially until there was no noticeable difference at the crown location. The direct extraction method fairly represented the linear extrapolation method for the SCF extraction. It was only short to the linear extrapolation method by 4% at the saddle location while higher by 4% at the crown location.

The shown results can fairly represent the SCFs from the experimental work as the least shown value was 18.3 from the direct extraction from first-order elements while the highest was 21.03 from the linear extrapolation method of second-order elements. Therefore, it was decided to use the highest value quoted by the linearly extrapolated SCFs from the second-order elements as the primarily SCF extraction method, as this was the method in line with experimental work and provided a SCF at the critical saddle location on the safe side.

### 4.6. Influence of Weld Profile Geometry

The variation in the SCF due to the weld profile modelling was investigated by modelling the tubular joints with an idealised “smallest” and “largest” accepted weld profiles and without the weld profile. The “smallest” accepted weld profile was the shortest acceptable weld leg length as per fabrication specifications and the “largest” was the most extended acceptable weld leg length as per fabrication specifications. For the model without the weld profile, the stresses at “a” and “b” were measured from a fictitious weld toe location similar to the lower bound condition.

The weld profile can affect the local stresses of the joints in two ways, the first is by the angle between the weld profile and chord surface as it affects the notch stresses, and the second is by changing the local stiffness of the intersection. The direct extraction method was affected by both the weld angle and weld size, while the linear extrapolation method, in theory, should not be affected by the weld profile angle, as the purpose of the methodology was to omit the notch stresses and capture only the deformation stresses.

It is not recommended to calculate the SCF using the direct extraction method for the FE model without the weld profile, as the effect of weld angle was missed. This model provides the highest SCF estimates using the linear extrapolation method since the sudden change in the angle between the brace and the chord formed a stress singularity.

The variation in the estimated SCF due to weld size (from lower bound to upper bound) was found to be less than 3.2% except for the direct extraction method from the first-order element where a variation of 9.8% was found.

The variation of the SCFs for models with different weld profiles was within the range of variation between different modelling techniques (18.3–21), see Table 6 and Table 7. Only the model with no weld profile pushes the maximum estimated SCF at the saddle outside these values to an SCF of 22.2. Hence, it seems more reasonable to model the weld and it is recommended to model both the lower bound and the upper bound of the weld profile, as the weld could end up as any of these, and use the highest SCF of these.

## 5. SCFs Comparison

**Figure 17:**
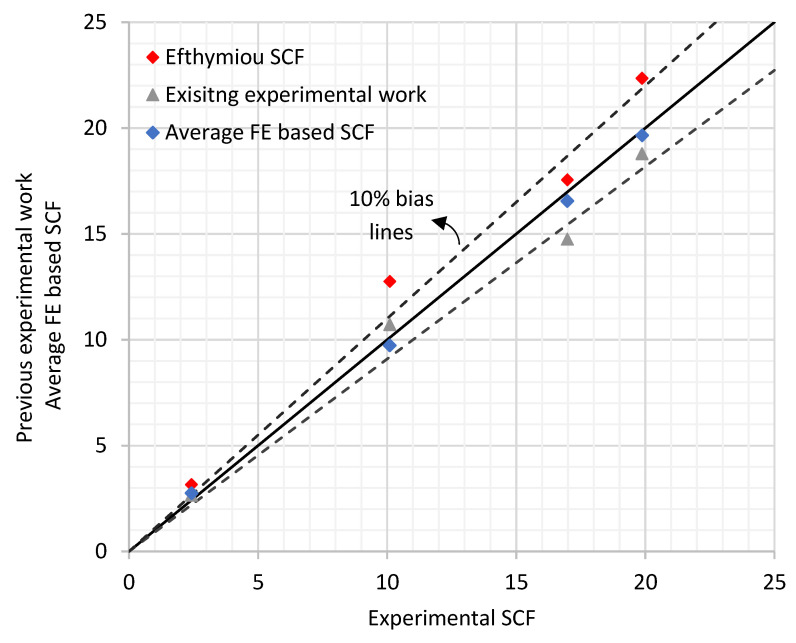
Present experimental mean SCF values against parametric Efthymiou, previous experimental work and FE based SCF values.

A comparison between the estimated SCFs is given in Table 8 and Figure 17. These methods include:

(1) Present experimental work.
(2) Previous experimental work [16,17,18,19].
(3) Range of valid finite element analyses.
(4) Efthymiou parametric formulae.

There is generally a good agreement between all the methods used to estimate the SCFs, where the Efthymiou equations form an upper bound.

**Figure 18:**
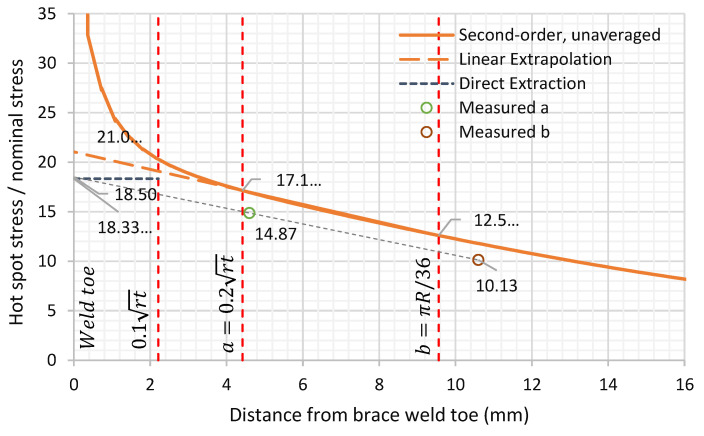
SCF based FEA compared to SCF from specimen DT 1—quadrant 3.

The FE based SCFs were modelled from the accurate weld cut segments of the first specimen from the third quadrant. As shown in Table 6, the results varied as per the selected modelling techniques. A comparison between the finite element based SCF and the experimental SCF of the same quadrant of same specimen is shown in Table 9 and Figure 18. The experimental results fell close to the lower bound SCF value of the finite element models for all the measurement on the joint circumference.

Estimation of SCFs by the aid of FEA was highly dependent on the user judgement. The SCFs values can change by the type of elements selected, mesh density and SCF extrapolation method. Four different SCF estimation methods from the FEA are shown in this paper, in which all of these models were valid as per codes and recommendations. Even though all meshes provide reasonable convergence levels, a variation in the SCF estimation of 15% was found between these different methods. Such a variation in the SCF when applied to the S–N curve with an inverse slope of three will yield a variation in the fatigue life of 52%, even though all these methods are valid.

## 6. Summary and Conclusions

Stress concentration factors for cruciform tubular joints of a chord and brace intersection (also called double T or X joints) were determined at different positions along the weld toe of the chord using detailed finite element analysis and from strain gauge measurements made during experimental testing of four representative joints. These SCFs were compared with SCFs determined from published parametric equations, including those by Wordsworth and Smedley and Efthymiou, the latter being the basis of those in the design codes ISO 19902 [1], API RP2A [3] and DNVGL-RP-C203 [4]. The intention of this study was to provide insight about the variations between SCFs from different methods so that users may take these into account when determining the fatigue life of the joint.

Detailed finite element analysis was undertaken to investigate the sensitivity of the SCF to:

(1) The mesh density,
(2) The choice of element type (1st order linear or 2nd order quadratic),
(3) The method for deriving SCF, either using direct extraction at the $0.1\sqrt{rt}$ location or from linear extrapolation of stresses to the weld toe,
(4) The range of weld profiles allowed by codes such as API RP2A [3].

The results showed a variation of the SCF with mesh size with the SCF tending to increase with the fineness of the mesh down to an element size of 0.36 mm. The analyses of different weld profiles showed that the SCF based on the maximum weld profile (according to API RP2A [3]) was higher than that for the minimum allowable weld profile, as the extra weld metal concentrates the loading of the chord at the weld toe. Using the finest mesh, the SCF at the saddle varied between:

(1) 18.33 using 1st order linear elements, direct extraction and the idealised largest accepted weld profile and;
(2) 21.03 using 2nd order quadratic elements, linear extrapolation and the idealised largest accepted weld profile.

The SCFs determined from strain gauge measurements at positions around the weld toes of the four nominally identical DT joints tested allowed the experimental variation of up to 16 nominally identical locations to be determined (i.e., four saddle points per joint). This database increased when SCFs at the crowns and two intermediate positions were calculated. From this the mean SCF, standard deviation and coefficient of variation (CoV) were determined at different positions of each joint and for the sample of four joints.

The mean experimental SCF at the saddle positions varied from 18.43 from specimen DT1 to 20.88 from specimen DT3, with an average SCF of 19.87 with a standard deviation of 1.72 and CoV of 8.7%. The variation can be seen to be relatively small and enables a statistical bias for design purposes. Although not directly related, these CoV values are within the range (5–10%) that probabilistic codes, such as DNVGL-RP-C210 [15], assumes for SCFs derived from a detailed FE analysis.

For two randomly selected specimens, the locations of the strain gauges were measured with an accuracy of 0.1 mm, reducing the average SCF at the chord saddle from 19.4 to 18.71 (bias of 3.7%) and reducing the CoV from 8.4% to 6.5%. If the inaccuracy in the location of the strain gauges can be assumed to be systematic also for the remaining two specimens, the average SCF of these experiments can be assumed to be 19.16 with a CoV of 8.4%.

In a further development, when specimen DT1 was sectioned, it was found to correspond with the largest weld profile allowed by API RP2A [3]. This enabled a comparison to be made with the detailed finite element analysis of this profile. Here reasonable agreement was obtained between the SCF found using 2nd order quadratic elements with a fine mesh and linear extrapolation (21.0) and the mean experimental SCF (19.9), providing a useful validation of the detailed finite element approach.

The SCF results of this study were then benchmarked against the standard empirical parametric equations used to calculate SCFs of tubular joints, including those by Wordsworth and Smedley and Efthymiou. These equations were themselves derived from experimental studies and finite element models and implemented in design codes such as ISO 19902 [1] and DNVGL-RP-C203 [4]. The use of the parametric equations was found to be well on the safe side and more detailed analysis could be beneficial if conducted using a fine mesh (e.g., 0.36 mm element) and 2nd order quadratic elements.

The following conclusions are therefore drawn from the study:

(1) SCFs determined using detailed finite element analysis were subject to variations depending on the mesh size, the choice of element type (linear or quadratic), the method for deriving the SCF (directly extracted or linearly extrapolated) and the weld profile modelled. In general, a higher SCF was obtained with a finer mesh, quadratic elements, linear extrapolation and a larger weld profile.
(2) The experimentally determined SCFs also show variations caused by the strain gauge positions and other inherent uncertainties. Comparison of the experimental SCFs with the SCF from detailed finite element analysis for a matching weld profile showed good agreement thereby validating the finite element approach.
(3) SCFs obtained from the parametric equations of Efthymiou [13] given in design codes ISO 19902 [1] and DNVGL-RP-C203 [4] were a reasonable upper bound to the variations in the values obtained by a detailed finite element analysis and experimentally in this study. These results provide continued support for the use of these equations in design. A detailed finite element analysis could be beneficial if small gains in the fatigue life need to be justified.

## Figures and Tables

**Table 1:** SCFs from the chord saddle to crown based on design Efthymiou SCFs and previous experimental work.

Location	Efthymiou	Average of Previous Experimental Work [16,17,18,19]
0° Saddle	22.35	18.82
22.5°	17.55	14.79
45°	12.76	10.76
90° Crown	3.16	2.69

**Table 2:** Experimental measured strain concentration factors (SNCF), FE validation and SCF correction factors from directional stresses perpendicular to the weld toe to principal stresses.

Location	0°—Saddle	22.5°	45°	90°—Crown
SNCF—directional (mean)	17.28	14.76	8.77	2.15
SNCF—directional (Std. dev.)	1.49	1.50	1.01	0.26
FE SCF—directional	16.67–18.34	13.37–15.02	7.87–9.03	2.28–2.28
FE SCF—maximum principal	19.12–21.03	15.35–17.22	9.06–10.38	2.68–2.68
Correction factor	14.7%	14.8%	15.2%	17.6%

**Table 3:** SCFs from experimental work (present data).

Specimen	Location	Saddle	* 22.5°	45°	Crown
DT 1	Number of points	4	8	8	-
	SCF (mean)	18.43	15.74	9.27	-
	Std. dev.	0.55	1.57	0.69	-
DT 2	Number of points	4	-	-	4
	SCF (mean)	20.36	-	-	2.53
	Std. dev.	1.06	-	-	0.31
DT 3	Number of points	3	7	7	-
	SCF (mean)	19.79	17.78	11.27	-
	Standard deviation	2.54	1.17	0.94	-
DT 4	Number of points	4	8	7	-
	SCF (mean)	20.88	17.50	9.87	-
	Std. dev.	1.93	1.89	0.87	-
Entire sample (DT 1, DT 2, DT 3 and DT 4)	Number of points	15	23	22	4
	SCF (mean)	19.87	16.98	10.10	2.53
	Std. dev.	1.72	1.73	1.17	0.31
	Coeff. of variation	8.7%	10.2%	11.6%	12.2%

* Angle measured from the chord saddle centre.

**Table 4:** Chord side saddle SCFs based on Mises and principal stresses for first-order elements.

Description	$\mathrm{Direct}\;\mathrm{Extraction}\;\mathrm{from}\;0.1\sqrt{rt}\;\mathrm{Location}$				Linear Extrapolation			
Element length	1.50	0.90	0.56	0.36	1.50	0.90	0.56	0.36
Elem. through-thickness	4	4	6	9	4	4	6	9
Principal (unaveraged)	16.55	16.91	17.74	18.33	19.11	18.16	18.73	19.12

**Table 5:** Chord side saddle SCFs based on Mises and principal stresses for second-order elements.

Description	$\mathrm{Direct}\;\mathrm{Extraction}\;\mathrm{from}\;0.1\sqrt{rt}\;\mathrm{Location}$				Linear Extrapolation			
Element length	1.50	0.90	0.56	0.36	1.50	0.90	0.56	0.36
Elem. through-thickness	4	4	6	9	4	4	6	9
Principal (unaveraged)	20.55	20.58	20.55	20.30	21.14	21.17	21.17	21.03

**Table 6:** Circumferential variation of extracted SCF by direct and linear extrapolation methods for first and second-order elements.

Location	$\mathrm{FEA}\;\mathrm{Direct}\;\mathrm{Extraction}\;\mathrm{from}\;0.1\sqrt{rt}\;\mathrm{Location}$		FEA Linear Extrapolation	
	First-Order Element	Second-Order Element	First-Order Element	Second-Order Element
0°—Saddle	18.33	20.31	19.12	21.03
22.5°	15.19	16.75	15.35	17.22
45°	9.31	10.4	9.06	10.38
90°—Crown	2.8	2.8	2.68	2.68

**Table 7:** SCFs for different weld profiles based on unaveraged principal stresses.

Weld Profile	$\mathrm{Direct}\;\mathrm{Extraction}\;\mathrm{from}\;0.1\sqrt{rt}\;\mathrm{Location}$		Linear Extrapolation	
	1st Order	2nd Order	1st Order	2nd Order
No Weld			20.2	22.2
Idealised smallest accepted weld profile	20.1	19.8	18.5	20.4
Idealised largest accepted weld profile	18.3	20.3	19.1	21.0

**Table 8:** Correlation between FEA, present and previous experimental work and Efthymiou SCFs.

Location	Present Experimental Work	Finite Element Analysis	Efthymiou	Previous Experimental Work
0° Saddle	19.87	18.3–21.0	22.35	18.82
22.5°	16.98	15.9–17.2	17.55	14.79
45°	10.10	9.06–10.4	12.76	10.76
90° Crown	2.53	2.7–2.8	3.16	2.69

**Table 9:** FEA based SCF compared to SCF from specimen DT 1—Quadrant 3 (corrected for location of “a” and “b”).

SCF	Saddle	22.5°	45°
Experimental mean	18.5	15.7	9.3
Finite Element analysis	18.3–21.0	15.9–17.2	9.06–10.4

## Data Availability

Data can be obtained from the first author upon reasonable request.
